# Cognitive Behavioural Therapy (CBT) for Managing Tinnitus, Hyperacusis, and Misophonia: The 2025 Tonndorf Lecture †

**DOI:** 10.3390/brainsci15050526

**Published:** 2025-05-19

**Authors:** Hashir Aazh

**Affiliations:** Hashir International Specialist Clinics & Research Institute for Misophonia, Tinnitus and Hyperacusis, London W1W 5PF, UK; info@hashirtinnitusclinic.com; Tel.: +44-0203-930-9523

**Keywords:** cognitive behavioural therapy (CBT), tinnitus, hyperacusis, misophonia, Tonndorf

## Abstract

Cognitive behavioural therapy (CBT) is an evidence-based intervention for managing distress associated with tinnitus, hyperacusis, and misophonia. This paper summarises key points from the 2025 Tonndorf Lecture presented at the third World Tinnitus Congress and the 14th International Tinnitus Seminar in Poland. The lecture addressed (1) the theoretical foundations of CBT for these conditions, (2) clinical evidence on CBT delivered by psychologists, audiologists, and digital self-help, and (3) the proportion of patients who may benefit from CBT. Research demonstrates that CBT can effectively reduce distress related to tinnitus, hyperacusis, and misophonia. Both psychologist- and audiologist-delivered CBT approaches have demonstrated significant improvements in reducing the impact of tinnitus, hyperacusis, and misophonia on patients’ quality of life, while guided internet-based CBT also demonstrates positive outcomes. Unguided internet-based CBT is also effective, though it faces challenges such as higher dropout rates. Despite these promising results, not all patients experience the same level of benefit. Some continue to experience distress even after completing CBT, highlighting the need for alternative or complementary interventions and ongoing support. This paper estimates that approximately 1 in 52 individuals with tinnitus require CBT, indicating that while tinnitus is relatively common, the need for intensive therapy is comparatively small. To enhance treatment outcomes, future research should compare the effectiveness of psychologist- and audiologist-delivered CBT, explore hybrid models that combine face-to-face and digital interventions, and address challenges with internet-based CBT, particularly for hyperacusis and misophonia. Furthermore, incorporating neuroimaging and physiological measures in future randomised controlled trials could provide objective insights into the neural mechanisms underlying symptom improvement, ultimately helping to refine CBT interventions. Identifying characteristics of non-responders to CBT may also guide the development of more tailored therapeutic approaches.

## 1. Introduction

Abraham Shulman and Barbara Goldstein organised the International Tinnitus Seminars (ITS), bringing together leading scientists of the 1960s and 1970s. In 1979, they convened eminent figures in the tinnitus field such as Jack Vernon, Ellis Douek, Harald Feldmann, Jean Marie Aran, Jack Pulec, and Juergen Tonndorf in New York. By initiating the ITS, Shulman and Goldstein strived to raise awareness about tinnitus [1] and, over the last half-century, have brought together scholars from various disciplines worldwide to share research on its causes, mechanisms, assessment, and treatment [2,3,4,5,6,7,8]. The ITS have been held in nine countries over five continents: three times in the United States (in 1979 and 1983 organised by Abraham Shulman and Barbara Goldstein and in 1995 organised by Jack Vernon and Gloria Reich), twice in France (in 1991 organised by Jean-Marie Aran and Rene Dauman and in 2005 organised by Rene Dauman and Frederic Bouscau-Faure), Germany (in 1987 and 2014, organised by Harald Feldmann and Birgit Mazurek, respectively), and Poland (in 2017 and 2025 organised by Henryk Skarżyński and Piotr Henryk Skarżyński), and once in the United Kingdom (in 1999 organised by Jonathan Hazell), Australia (in 2002 organised by Pam Gabriels), Sweden (in 2008 organised by Kajsa-Mia Holgers and Gunilla Jansson), Brazil (in 2011 organised by Tanit Sanchez), and Kyrgyzstan (in 2023 organised by Henryk Skarżyński and Piotr Henryk Skarżyński). In 2017, the World Tinnitus Congress (WTC) was established alongside the ITS as an initiative by Henryk Skarżyński and Piotr Henryk Skarżyński. This expansion aimed to broaden the scope of ITS beyond a seminar format, transforming it into a global, intercontinental congress.

The organisers of the second ITS in 1983 invited Juergen Tonndorf as their guest of honour [9,10]. Tonndorf was a prominent auditory scientist, otologist and research professor who published extensively on various topics ranging from cochlear mechanics to the inhibition of tinnitus and was the first to propose the analogy between tinnitus and pain [11]. He completed his medical training in Kiel University, Germany, in 1938 and received a PhD from Heidelberg University in 1945 [10]. Tonndorf served as a physician and captain in the submarine corps of the German Navy during World War II before moving to the United States in around 1947. He was highly valued among researchers and was considered as the leading scientist in the tinnitus field at the time. After his passing in 1989, the Tonndorf Lectures were introduced at the ITS in his honour. These lectures provide an opportunity to reflect on advancements in tinnitus research and explore future directions [12,13,14].

I was honoured to deliver the Tonndorf Lecture at the 14th International Tinnitus Seminar and 3rd World Tinnitus Congress, held in Poland from 13 to 15 April 2025 [15]. My lecture examined the theoretical foundations of cognitive behavioural therapy (CBT) and its application in the management of tinnitus and sound intolerance conditions, including hyperacusis and misophonia. The key concepts and arguments from that lecture are summarised in this article. Tinnitus refers to the perception of sound in the absence of an external stimulus, while hyperacusis and misophonia involve reduced tolerance to certain sounds (known as trigger sounds)—either perceiving them as excessively loud (hyperacusis) or intensely irritating/annoying (misophonia) [16,17,18]. This paper is structured into three main sections: (1) the theoretical foundations of CBT, (2) research evidence on the clinical application of CBT—delivered by psychologists, audiologists, or digital self-help—for managing tinnitus, hyperacusis, and misophonia, and (3) the proportion of patients who may benefit from CBT.

## 2. Theoretical Underpinnings of CBT for Tinnitus, Hyperacusis and Misophonia

### 2.1. Emotional Response to Tinnitus or Trigger Sounds

Individuals with tinnitus, hyperacusis, misophonia report experiencing a variety of emotions in response to perceiving their tinnitus or when they are exposed to the triggering sound(s) comprising feelings of unhappiness, anxiety, irritability, anger, depressed mood, annoyance, helplessness, despair, hopelessness, panic, rage, impatience, disgust, fear, or violation, or feelings of being trapped, stressed out, upset, threatened, afraid, hostile, hateful, and jittery [19,20,21,22,23,24,25,26]. Imaging studies have shown that certain brain areas that are responsible for emotions (e.g., the amygdala, limbic network, and insula) exhibit alterations in their activity and their functional connectivity in people with distressing tinnitus as well as in people with hyperacusis and misophonia (when exposed to the triggering stimuli) [27,28,29]. In other words, biological evidence supports the self-reported emotional responses associated with tinnitus perception or exposure to trigger sounds. When tinnitus or trigger sounds (in the case of hyperacusis or misophonia) evoke emotions, they are considered emotionally significant stimuli. This enhances their processing in the brain compared to neutral stimuli, making them more likely to be perceived and remain the focus of attention [30,31,32,33]. This may explain the challenges patients face, such as difficulty redirecting attention away from tinnitus [34], struggling to ignore everyday sounds in hyperacusis [35], and finding it hard to shift focus away from trigger sounds in misophonia [36]. Additionally, the emotional associations with tinnitus or trigger sounds can hinder the process of habituation [37,38,39,40]. Habituation is a natural mechanism that reduces an individual’s response to repeated stimuli, helping to make tinnitus less noticeable, allowing for better ignoring of trigger sounds in misophonia and hyperacusis [41,42,43]. Therefore, understanding the underlying mechanisms responsible for emotional responses to tinnitus and trigger sounds—and how to modify them—is crucial for developing effective rehabilitative therapies.

### 2.2. Cognitive Theory and the Origin of Emotional Disturbances

Mathematical modelling (i.e., regression models and mediation analysis) has been used to estimate the direct and indirect relationship between psychophysical characteristics of tinnitus and/or sound intolerance and the emotional disturbances experienced by the patients [44,45]. For example, Aazh and Moore [44] performed a mediation analysis on data from 620 patients with tinnitus and/or hyperacusis and reported that the tinnitus loudness as measured via the visual analogue scale (VAS) [46] was not directly associated with their depressive symptoms as measured via the depression subscale of the Hospital Anxiety and Depression Scale (HADS-D) [47]. Their results suggested that the relationship between tinnitus loudness and depression was indirect, mediated by the impact tinnitus had on the sufferer’s life. They reported a similar result for people with hyperacusis as the severity of depression was not directly linked with the actual uncomfortable loudness levels (ULLs) but was determined by the effect of hyperacusis on their life as measured via the Hyperacusis Questionnaire (HQ) [35]. Numerous studies suggest that the impact of tinnitus or hyperacusis on a patient’s life is a key predictor of depression among sufferers [48,49,50]. A patient’s subjective report of the impact of tinnitus or hyperacusis on their life is a psychological process that depends on their interpretation of daily activities and overall quality of life. In other words, an individual’s thought process regarding the causes of tinnitus, the source of trigger sounds, and the perceived impact of tinnitus or exposure to trigger sounds predicts their emotional response. This is consistent with cognitive theory [51]. Cognitive theory postulates that an individual’s thought process may be influenced by certain errors in judgment, which can be exacerbated by emotional disturbances, increasing both their frequency and intensity [51,52,53,54]. For example, the individual may be jumping to a conclusion or catastrophizing [55] by thinking that “I can no longer cope with tinnitus” [34], “my tinnitus will lead to a nervous breakdown” [56], “the noise will drive me crazy” [57,58], “my whole life will be affected by sound issues” [21], “people think I’m crazy because of my reaction to sounds” [26], “my sound intolerance will make it difficult to do the things I used to enjoy” [50] or ”my sensitivity to sound will eventually isolate me” [59]. Thinking that “because of my tinnitus, I feel that I have a terrible disease” [34] or “my tinnitus is the result of a tumour, brain haemorrhage, or becoming deaf” [58] exaggerates the severity of its underlying cause. Thinking that “it is my own fault that I have developed tinnitus” [48] or “the way that I react to certain sounds proves that deep inside I am just a bad person” [59] irrationally internalises the cause of the problem, leading to self-blame. Thoughts such as “people who make noises are disrespectful, bad-mannered, selfish, and thoughtless” [59] or “people make sounds on purpose just to upset me” [26] reflect irrational judgments and misunderstanding about the personality and intentions of other people. Some thoughts reflect reasoning based on emotions as opposed to reasoning based on facts [60]. For instance, the patient may think that “because I feel helpless then it means that tinnitus will make my life unbearable and that I cannot cope with it” [57] or “because I feel frightened and anxious when hearing certain sounds, it means that exposure to such sounds must be harmful and damage my hearing, worsen my tinnitus or make me more sensitive to sound” [61], or a patient with misophonia may think that “because I feel disgusted or angry when hearing people’s chewing noises, this means that the people who make the noises are bad mannered and that I am being treated unfairly” [62]. According to cognitive theory, a patient’s avoidance or ritualistic behaviours stop them from learning whether their thoughts and predictions about the impact of tinnitus and/or sound are realistic or they reflect a misunderstanding. This leads to the maintenance of the distress caused by such thoughts. For instance, patients with tinnitus often avoid silence due to the fear of being unable to cope if fully exposed to tinnitus [63]. Some may engage in substance abuse to feel better [64] or avoid certain social environments, fearing that loud noises could worsen their condition [58]. Patients with hyperacusis frequently report avoiding leaving their homes, refraining from using public transportation, and overusing ear protection (such as earmuffs or earplugs) out of fear of exposure to loud sounds [35,50,65]. Similarly, patients with misophonia exhibit behaviours such as playing background music to mask bothersome trigger sounds, criticising individuals who produce the noise, leaving the room or dinner table, staring at noise-makers to signal their discomfort, shouting to stop the noise, or even mimicking the offending sounds [66,67]. Research indicates that while avoidance and ritualistic behaviours may provide short-term relief, they often contribute to long-term distress associated with these conditions rather than alleviating it [68,69,70,71].

### 2.3. CBT Conceptual Models for Tinnitus, Hyperacusis and Misophonia

**Figure 1:**
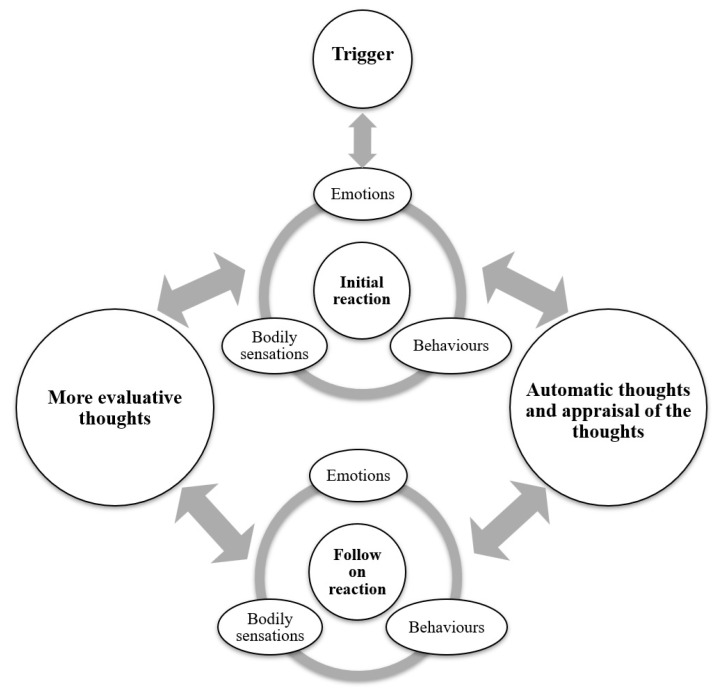
Transdiagnostic CBT model for tinnitus, hyperacusis and misophonia.

In CBT, conceptual models are used to understand the cognitive and behavioural processes that give rise to or maintain the patient’s distress. These models help demonstrate the interconnections between perceiving tinnitus or trigger sounds, thoughts, feelings, and behaviours. CBT then guides the patient in recognising and modifying these cognitive processes and behavioural patterns to break the vicious cycle of tinnitus-, hyperacusis-, or misophonia-induced anxiety and annoyance. Here, some of the published CBT models for tinnitus, hyperacusis and misophonia are briefly discussed [62,72,73,74,75,76,77,78,79]. Cima et al. [80] and Kleinstäuber et al. [81] proposed a model suggesting that when individuals catastrophically misinterpret tinnitus, they experience tinnitus-related fear, leading to avoidance behaviours, heightened awareness, and disability. This cycle ultimately enhances the perception of tinnitus. Conversely, if individuals perceive tinnitus as a benign signal, they continue their normal activities without avoiding tinnitus, which facilitates recovery [73,82]. McKenna et al. [74] proposed a model suggesting that intrusive negative automatic thoughts, distorted perceptions, maladaptive beliefs, and safety behaviours contribute to increased arousal and distress. This heightened distress leads to selective attention, further monitoring, and enhanced detection of tinnitus. In turn, this cycle reinforces and strengthens negative automatic thoughts and distorted perceptions related to tinnitus. Gregory et al. [77] proposed a model for misophonia, suggesting that individuals with the condition experience an acute reaction—encompassing thoughts, emotions, and bodily sensations—when exposed to or anticipating trigger sounds. This heightened response increases their focus on these sounds, reinforcing or shaping certain beliefs about themselves, others, and the future. Consequently, cognitive, emotional, and behavioural dysregulation intensifies, lowering the threshold at which sensory cues become distressing. This, in turn, amplifies sensitivity to sensory stimuli and impairs the ability to filter out irrelevant, repetitive cues. Webber and Storch [78] proposed a CBT model for misophonia, suggesting that trigger sounds evoke a negative emotional reaction, which is then reinforced through behavioural responses that provide temporary relief but ultimately sustain the cycle. Aazh et al. [62,79] proposed a transdiagnostic CBT model to conceptualise distressing tinnitus, hyperacusis, and misophonia (Figure 1). Their model begins with the initial reaction to the perception of tinnitus or trigger sounds, which includes an emotional response, bodily sensations, and certain behaviours. This initial reaction is considered automatic or an involuntary reflex (see Table 1 for examples). Following this, automatic thoughts and their appraisal shape a secondary, or follow-on, reaction, which also involves emotions, bodily sensations, and behaviours. Unlike the initial reaction, which occurs without preceding thoughts, follow-on reactions are directly influenced by an individual’s automatic thoughts and their interpretations. This process leads to further evaluative thoughts, creating a feedback loop that reinforces distress and exacerbates symptoms. Most CBT models described here are conceptual frameworks that often require adjustments and customization for each individual patient.

Table 1 illustrates how avoidance and maladaptive thought patterns contribute to the maintenance of distress, highlighting the need for targeted CBT interventions to break this cycle. CBT is delivered through a structured, collaborative process that supports individuals in identifying and modifying these maladaptive patterns. Treatment begins with psychoeducation to help the patient understand the cognitive behavioural processes that lead to the distress they are experiencing as a result of their tinnitus or sound intolerance, and how to identify and modify these processes. This is followed by guided discovery [97] and Socratic dialogue [98] to help individuals recognise the automatic thoughts, beliefs, and behaviours maintaining their distress. Behavioural experiments are used to test out predictions (e.g., “If I go out without earplugs, I will panic”), while cognitive restructuring helps patients develop more balanced appraisals (e.g., “Discomfort doesn’t mean danger”). The goal is to reduce avoidance, reframe unhelpful cognitions, and promote emotional regulation through experiential learning and reflective practice. The therapeutic relationship provides a foundation for change, offering validation, empathy, and a safe space for experimentation.

## 3. Research Evidence on CBT for Management of Tinnitus, Hyperacusis and Misophonia

### 3.1. Effectiveness of Psychologist-Delivered CBT in Managing Tinnitus

Ten out of the eleven published clinical practice guidelines for tinnitus recommend the use of CBT [99,100]. There are several reviews of the research literature assessing the effect of CBT on tinnitus distress [101,102,103,104,105,106]. Martinez-Devesa et al. [104] reviewed the literature up to May 2010 and included eight randomised controlled trials (RCTs) including 468 participants to their analysis. They concluded that a significant reduction (improvement) in tinnitus impact and depression was achieved by patients who received CBT compared to the control groups. Hesser et al. [105] also reviewed RCTs published until 2010 but they included fifteen studies with total of 1091 participants in their analysis. Their results showed that patients who received CBT achieved greater reduction in tinnitus impact, which was statistically significant with mean effect sizes (ESs) of 0.70 (when compared to passive control groups, i.e., patients receiving no intervention) and 0.44 (when compared to active control, i.e., patients receiving treatments other than CBT). Fuller et al. [102] assessed studies published until November 2019 and included twenty-eight studies with a total of 2733 participants in their analysis. They concluded that CBT may be effective in reducing the impact of tinnitus on the patient’s life. Curtis et al. [101] reviewed the studies that assess the effect of CBT on insomnia in people with tinnitus published until 2019. They included five RCTs in their analysis and concluded that CBT can significantly improve sleep in adults with tinnitus.

Although one study reported that approximately 75% of individuals who underwent CBT for tinnitus found the treatment both acceptable and beneficial [107], the evidence regarding acceptability remains limited. For example, while one study suggested lower acceptability among veterans [108], this single finding is insufficient to draw firm conclusions, and further research is needed to assess acceptability across different populations more systematically. The CBT interventions in the studies that were assessed in the above-mentioned systematic reviews were largely conducted by psychologists, psychology assistants/students, or other professionals in mental health settings. Fuller et al. [109] reported that an intervention model combining care from audiologists and psychologists led to clinically meaningful improvements across various outcome measures, with the CBT component delivered by psychologists.

### 3.2. Effectiveness of Audiologist-Delivered CBT for Tinnitus Management

Between 2018 and 2025, more studies emerged assessing the outcome and practical implications of audiologist-delivered CBT [110,111,112,113,114,115,116,117,118,119]. Aazh and Moore [112] reported that 36 patients who received audiologist-delivered CBT in a UK National Health Service (NHS) Audiology Department showed a significant reduction in tinnitus impact, as measured by the Tinnitus Handicap Inventory (THI) [120], with ESs of 1.13 after 6 weeks. Another study by Aazh et al. [110] evaluated the acceptability of audiologist-delivered CBT for tinnitus in a group of 31 patients. Participants were asked how acceptable it was to receive CBT in an audiology department rather than a mental health setting within the NHS, using a scale from 1 (not acceptable at all) to 10 (very acceptable). The median rating was 10/10 (SD = 1.1), with 97% of patients rating it 7 or higher. Patients also assessed the effectiveness of CBT components in managing tinnitus and hyperacusis on a scale from 1 (not effective) to 10 (very effective). Median ratings were 9/10 (SD = 1.5) for behavioural experiment (BE) and Diary of Thoughts and Feelings (DTF), 9/10 (SD = 1.14) for psychoeducation (learning about the CBT model), and 10/10 (SD = 1.3) for therapist’s empathic listening. While no significant differences were found between BE, DTF, and psychoeducation, empathic listening was rated significantly higher than all three. Comparing tinnitus patients with and without hyperacusis (HQ ≥ 22, *n* = 18; HQ < 22, *n* = 13), those with HQ ≥ 22 rated BE (*p* = 0.04), DTF (*p* = 0.026), and psychoeducation (*p* = 0.002) significantly more effective.

A recent study found that patients who received audiologist-delivered CBT via video calls experienced a significant reduction in tinnitus impact (n = 12). The therapy programme consisted of 14 one-to-one online sessions, with the first 10 conducted weekly, followed by progressively longer intervals of 2 weeks, 1 month, 3 months, and 6 months. Each session lasted between 45 and 60 min. Self-report questionnaires were administered before the first CBT session and again at the final session (session 14), which also served as a 6-month follow-up assessment. The study measured tinnitus impact using the Tinnitus Impact Questionnaire (TIQ) [121] and THI, reporting large ESs of 2.6 and 2, respectively [113]. Prior to treatment, 100% of patients had severe tinnitus impact based on the TIQ, and 92% reported severe impact with 8% reporting moderate impact based on the THI. Following CBT, these numbers dropped substantially: 37.5% of patients still experienced tinnitus impact according to the TIQ (25% severe impact, 12.5% slight impact), while 55.5% continued to report an impact based on the THI (33% slight impact, 22% severe impact). Notably, 75% of patients had consulted mental health professionals before starting audiologist-delivered CBT, and 33% were already receiving psychiatric medication or psychological therapies in addition to CBT for tinnitus. However, this study did not determine whether taking medications or undergoing additional counselling had an impact on the outcomes, as the sample size was too small to draw definitive conclusions. Future studies with larger samples are needed to explore the potential effects of these factors.

### 3.3. Effectiveness of Guided and Unguided Internet-Based CBT for Tinnitus Management

Beukes et al. [122] conducted an RCT and reported that patients who received internet-based CBT (iCBT), guided by audiologists, showed a greater reduction in tinnitus impact as measured via the Tinnitus Functional Index (TFI) [123,124] compared to patients who received face-to-face tinnitus management counselling (non-CBT counselling) with an ES of 0.45. They reported that 52% of patients who received iCBT (n = 46) achieved a clinically significant improvement, with TFI scores falling below the threshold for requiring intervention (score < 25). In another RCT, Beukes et al. [125] reported a larger ES of 0.7, showing that guided iCBT was more effective than a control group receiving only weekly monitoring (n = 146). Uncontrolled studies with a repeated-measures design found that patients who received iCBT (n = 104 and 132) (guided by audiologists) maintained significant tinnitus relief even one-year post-treatment, with an ES of 1.06 on the TFI [126,127]. After completing iCBT, 41% of patients achieved TFI scores below the threshold for requiring intervention (<25).

iCBT as a self-help programme without the involvement of a clinician (unguided) has also been shown to have potential to help people manage their tinnitus [128,129]. Aazh et al. [129] surveyed 28 tinnitus patients who completed unguided iCBT. When asked how effective the programme was in managing their tinnitus (0 = “not effective at all” to 10 = “very effective”), the median response was 6/10 (SD = 2.6). Patients also rated their ability to manage tinnitus differently post-intervention at 7/10 (SD = 3.0). The study reported an ES of 0.6 for improving self-efficacy (4C questionnaire) [130] and 0.62 for reducing distress as measured via screening for anxiety and depression in tinnitus (SAD-T) [131]. The number of participants with SAD-T scores greater than 4, indicating symptoms of anxiety and depression requiring psychological interventions, decreased from 16 before treatment to 9 after treatment.

Walter et al. [132] conducted an RCT investigating the efficacy of smartphone-based unguided CBT among 187 patients with chronic tinnitus over a 9-month period. They reported that compared to a passive control group (waiting list), patients in the intervention group showed a greater reduction in tinnitus impact as measured via the German tinnitus questionnaire (TQ) [133], with an ES of 1.38, and a greater reduction in depression score as measured via the German patient health questionnaire (PHQ-9) [134], with an ES of 0.68. Patients generally had improved to lower-severity categories; for example, after 9 months, about 63% of patients were characterised as having mild tinnitus severity (TQ ≤ 30). While unguided iCBT is effective, dropout rates tend to be higher compared to face-to-face or guided iCBT programmes [135]. For example, dropout rates of approximately 51% have been reported for unguided iCBT [132,136], compared to dropout rates ranging from 21% to 32% for face-to-face CBT [104,112]. Aazh and Danesh [137] surveyed 41 healthcare professionals on the use of iCBT for tinnitus; 87% preferred unguided iCBT as a complementary intervention to their existing services and 56% were open to using iCBT as a standalone treatment. Guided iCBT for tinnitus, delivered by audiologists, has also been shown to improve hyperacusis symptoms too. Compared to a passive control group (weekly monitoring), it achieved an ES of 0.3 [125], with improvements sustained one year after treatment (ES = 0.26) [127]. However, currently there is no published study assessing the efficacy of iCBT dedicated to hyperacusis or misophonia management, although there are some studies in progress [138,139].

### 3.4. Effectiveness of CBT Delivered by Audiologists, Psychologists, and Occupational Therapists in Managing Hyperacusis

One RCT assessed the effect of psychologist-delivered CBT compared with a passive control group (patients on a waiting list) on hyperacusis (n = 62) [140]. Their results showed that compared to a control group, patients who received CBT exhibited a greater reduction in their hyperacusis symptoms as measured via ULLs and HQ, with ESs of 0.69 and 1.13, respectively. In a retrospective analysis of clinical data accumulated over a 10-year period, Nolan et al. [141] reported that hyperacusis symptoms as measured via the Geräuschüberempfindlichkeits-Fragebogen [142] questionnaire for 268 patients improved with an ES of 0.82 following an intervention encompassing CBT, physical therapy, musical and hearing therapy, ear acupuncture and weekly meetings with a psychiatrist (treatment protocol was applied equally to all subjects and lasted for 6–8 weeks).

In a retrospective service evaluation survey, Aazh and Moore [112] reported that patients who received audiologist-delivered CBT for hyperacusis (n = 43) showed improvements in HQ scores, with an ES of 0.93 after six weeks. Another study by Aazh et al. [110] found that patients with both hyperacusis and tinnitus reported greater benefits from audiologist-delivered CBT in managing their symptoms compared to those with tinnitus alone (n = 31). A case study by Carson et al. [70] reported that an 11-year-old boy with autism and hyperacusis demonstrated improved ULLs and daily sound tolerance following a CBT programme delivered by occupational therapists. More recently, Aazh et al. [113] reported that patients with hyperacusis who received audiologist-delivered CBT via video calls (n = 11) showed significant improvements across multiple self-report measures, including the HQ, Hyperacusis Impact Questionnaire (HIQ) [131], Inventory of Hyperacusis Symptoms (IHS) [50], and the Sound Sensitivity Symptoms Questionnaire (SSSQ) [16], with an ES of 0.9 across all measures [113]. Before treatment, 91%, 69%, 83%, and 85% of patients had abnormal scores on the HIQ, HQ, IHS, and SSSQ, respectively. Following CBT, these figures dropped to 22%, 33%, 37.5%, and 22%. It is worth noting that 77% of the patients had consulted mental health professionals before starting CBT, and 38.5% were taking psychiatric medication or receiving psychological therapies. These interventions targeted underlying psychological issues, while CBT specifically aimed to manage hyperacusis-related distress. The study assessed whether the use of medications or counselling influenced treatment outcomes, but the results were not statistically significant, possibility due to the small sample size. For the participants who were receiving psychiatric medication or psychological therapies—largely for anxiety and mood disorders rather than for hyperacusis or tinnitus—these interventions may have contributed to their overall improvement. However, to the authors’ knowledge, there is currently no evidence that such treatments directly improve hyperacusis. Therefore, the observed benefits of audiologist-delivered CBT should be interpreted with caution, and future controlled studies are needed to isolate its specific effects.

### 3.5. Effectiveness of Audiologist- and Psychologist-Delivered CBT for Managing Misophonia

Several case studies have documented the benefits of CBT for patients with misophonia [75,93,143,144,145]. A study involving 90 patients found that 48% experienced a significant reduction in misophonia symptoms following CBT [146]. The first RCT on CBT for misophonia, conducted by Jager et al. [147] in Amsterdam, reported that patients who received CBT (n = 46) showed greater reductions in misophonia severity, as measured by the Amsterdam Misophonia Scale—Revised (AMISOS-R) [92], with an ES of 1.97 compared to the passive control group (waiting list). These improvements were maintained 12 months post-intervention. In this study, CBT was delivered by psychologists in a group setting. Preliminary findings from a pilot study by Lewin et al. [76] also suggest that CBT can improve misophonia symptoms (n = 4). A retrospective service evaluation by Gregory et al. [77] examined 19 patients who received CBT from a specialist psychology service and found a reduction in misophonia severity, with an ES of 1.63 as measured by the Misophonia Questionnaire (MQ) [148]. Additionally, Aazh et al. [113] reported that nine patients with misophonia who received audiologist-delivered CBT via video calls (n = 9) demonstrated significant improvements in their symptoms, as measured by the Misophonia Impact Questionnaire (MIQ) [149], with an ES of 0.73. Among patients with misophonia, 78% had consulted mental health professionals and 44% were taking psychiatric medication or receiving psychological therapies to address underlying psychological issues, while CBT specifically aimed to manage misophonia-related distress. Before treatment, 78% of patients experienced significant distress according to the MIQ, which reduced to 22% after CBT.

## 4. Is CBT Necessary for Everyone with Tinnitus and Sound Intolerance?

### 4.1. Epidemiological Studies on the Prevalence of Tinnitus, Hyperacusis, and Misophonia Requiring Treatment

An epidemiological study analysing data from 75,764 adults estimated that 9.6% of the general population in the United States experienced tinnitus within a 12-month period [150]. Among those with tinnitus, only 7.2% considered it a “big” or “very big” problem, while 20.2% reported it as a moderate problem. The remaining 72.6% described their tinnitus as a “small” problem or were not bothered by it at all [150]. This is consistent with findings from a study in England that examined data from 48,313 individuals. In this study, 10.1% reported persistent tinnitus (lasting more than five minutes), 4.4% found their tinnitus to be “severely” or “moderately” annoying, and only 0.5% said it severely impacted their lives. The majority reported that tinnitus did not cause them distress [151].

Not everyone with tinnitus perceives it as problematic enough to seek treatment [152]. In a study by Rademaker et al. [153], it was found that 33.3% of individuals with tinnitus (72 out of 216) had sought help. Those who sought help typically had worse hearing thresholds, experienced a greater impact from tinnitus, and exhibited more severe somatization symptoms (e.g., pains in the heart or chest, nausea or upset stomach, and hot or cold spells) compared to those who did not seek treatment [153,154]. However, not all patients with tinnitus who seek medical advice are considered eligible for referral to ENT or audiology services. An analysis of data from 125,430 tinnitus patients in the UK revealed that only 22.26% were referred to ENT or audiology, identifying this group as having “clinically significant tinnitus” [155]. Furthermore, even among those referred to specialist audiology services, not all are considered candidates for CBT after further assessment through validated questionnaires or clinical interviews. Aazh and Moore [111] evaluated 226 patients referred to a specialist audiology clinic in the UK for tinnitus and/or hyperacusis. All patients had previously consulted their general practitioner (GP), and 85% had also seen an ENT specialist before being referred to audiology. According to the definition proposed by Martinez et al. [155], these patients would be classified as having “clinically significant tinnitus”. However, Aazh and Moore [111] reported that 85 out of 266 patients (32%) did not require any further intervention for tinnitus or hyperacusis. Despite being referred by their GP, these patients were not distressed by their symptoms and primarily sought reassurance regarding potential underlying medical conditions. Consistently, they scored low on validated self-report questionnaires assessing tinnitus, hyperacusis, and insomnia, with mean scores of 22% on the THI, 9.7/42 on the HQ, and 7.4/28 on the Insomnia Severity Index (ISI) [156]. The remaining 181 patients attended a second session and, based on an in-depth clinical interview given in that session, 124 (47%) were identified as experiencing tinnitus- or hyperacusis-related distress and were offered CBT. This interview method, developed by Aazh and Moore [112], was based on qualitative methods for health research [157] and exploratory strategies used in motivational interviewing in healthcare [158]. The in-depth interview aimed to determine whether tinnitus and/or hyperacusis disrupted daily life or affected mood and to distinguish between distress directly linked to these conditions and distress caused by other psychological, medical, or social factors. Patients were guided to reflect on their daily activities and evaluate whether their tinnitus or hyperacusis had a meaningful impact. If it was determined that their symptoms did not significantly disrupt their life or mood, CBT was deemed unnecessary. Interestingly, there was no statistically significant difference in the mean questionnaire scores (THI > 58, HQ > 18, and ISI > 14) between the 57 patients who were discharged after the in-depth interview and the 124 who were offered CBT. This suggests that even validated psychometric instruments may not reliably distinguish between patients whose distress is directly related to tinnitus or hyperacusis, those whose distress arises from other factors, and those who are not distressed at all. In total, 17 out of these 57 patients appeared to have emotional disturbances unrelated to their tinnitus and/or hyperacusis, likely stemming from an underlying psychological disorder. Consequently, they were referred for further psychological evaluation and potential treatment. For the remaining 40 patients, both the patient and the audiologist agreed that their current management strategies were effective, and no formal intervention was deemed necessary. In other words, their tinnitus and/or hyperacusis did not significantly disrupt their daily activities or negatively affect their mood. Finally, not all patients offered CBT chose to proceed with treatment. Of the 124 patients recommended for CBT, only 68 (26% of the original 266 subjects) accepted the offer, while 56 declined. Among those who declined, the majority (43 patients) reported that the assessment process and clinical interview helped them recognise they were managing their symptoms adequately and did not require further intervention. For the remaining 13 patients, the reasons for declining treatment were transport problems, insufficient time, and prioritising other health issues. Overall, only 26% of those initially classified as having “clinically significant tinnitus” and referred to audiology ultimately proceeded with CBT, despite it being freely available through the NHS.

To sum up, while 10.1% of the population (1 in 10 people ≈6.9 million) experience persistent tinnitus [151], data from a non-UK study suggests that only 33.3% may seek help (1 in 29 people ≈3.4% of UK population ≈2.3 million) [153]. Generalising from the two studies reviewed here, of those who seek help, 22.26% are referred to ENT or audiology (1 in 132 people ≈0.75% of UK population ≈509,000) [155], and ultimately, just 26% proceed with CBT (1 in 504 people ≈0.2% of UK population ≈133,000) [111]. These figures indicate that although tinnitus is common, only a small proportion of those affected require CBT to manage their symptoms.

### 4.2. Hyperacusis and Misophonia as Disorders

Not everyone who is sensitive to certain sounds can be classified as having a sound intolerance disorder. People’s reactions to sound vary due to multiple factors, including age, gender, hearing status, fatigue, prior sound exposure, lifestyle, social status, overall health, medication use, noise levels in their living environment, duration of residence, and psychological well-being [59,159,160,161,162,163]. Some individuals are naturally more sensitive to noise than others. Noise sensitivity is considered a personality trait linked to one’s attitude and self-perceived vulnerability to noise and is a key predictor of noise annoyance [164]. In other words, individuals with higher noise sensitivity are more likely to report being disturbed by noise and experiencing a reduced quality of life due to environmental sounds [165].

A study in New Zealand found that approximately 50% of participants from the general population were moderately noise-sensitive [160]. This suggests that being disturbed by certain sounds is not necessarily an indication of a disorder or abnormality, as it appears to be a common experience among half of the population. Similarly, Bigras et al. [166] asked 59 individuals who self-identified as hypersensitive to loud sounds to complete the HQ. Based on their scores, only 30.5% (18 out of 59) met the criteria for hyperacusis (HQ score ≥ 22) [167]. This indicates that not everyone who perceives everyday sounds as excessively loud or unpleasant has hyperacusis.

A similar pattern is observed with misophonia. Naylor et al. [168] conducted an online survey among medical students at the University of Nottingham, UK. Their results showed that 49.1% of 336 respondents exhibited misophonia traits based on the Amsterdam Misophonia Scale (A-MISO-S) [36]. This suggests that feeling irritated or angered by certain sounds, such as eating noises, is a common experience for nearly half of the study population. Therefore, it would be unreasonable to classify it as a disorder in all cases. Another study, which surveyed 772 participants from the general UK population, found that around 40% reported feeling disgusted when hearing loud chewing, while approximately 50% were irritated by sounds such as dog barking, tapping, slurping, snoring, repetitive sniffing and coughing, loud breathing, and engine noise [59]. However, when assessed using the S-Five questionnaire [66], only 18.4% (142 out of 772) experienced misophonia to a degree that significantly impacted their daily lives. These findings suggest that while many individuals report sensitivity to certain sounds, only a subset of them experience symptoms severe enough to be classified as hyperacusis or misophonia. Sensitivity to noise is common, but it does not necessarily indicate a clinical disorder. Unlike tinnitus, there is currently insufficient population-level research to reliably estimate the proportion of individuals with hyperacusis or misophonia who seek or receive treatment, which limits direct comparison and underscores the need for improved epidemiological data in these emerging fields.

## 5. Discussion

### 5.1. Improving CBT Models

CBT relies on conceptual models to explain the cognitive and behavioural processes that contribute to the distress experienced by patients with tinnitus, hyperacusis, and misophonia discussed [62,72,73,74,75,76,77,78,79]. These models illustrate how the perception of tinnitus or triggering sounds interacts with thoughts, emotions, and behaviours to create cycles of distress. While these frameworks guide clinical interventions, they remain largely theoretical and require empirical validation using patient data. One approach to model validation involves path analysis, a statistical method that assesses the direct and indirect relationships between variables contributing to distress [169]. Future research should examine the strength of these relationships to refine CBT models and assess their ability to explain the mechanisms underlying distress. Longitudinal studies could help determine whether specific model components influence the development or reduction of distress, thereby improving their clinical relevance. Additionally, research should compare the applicability of CBT models in individuals who experience distressing symptoms versus those who do not [170]. For example, Handscomb et al. [171] surveyed 342 individuals with tinnitus, measuring each construct within a specific CBT model. Using path analysis and established fit criteria, they assessed the model’s goodness of fit. Their findings suggest that different configurations of the CBT model may be more suitable for different groups of people with tinnitus, primarily differing in the placement of tinnitus magnitude and the inclusion or exclusion of control beliefs. Given these findings, further research should explore the relative influence of the psychophysical characteristics of tinnitus or trigger sounds versus cognitive factors in shaping distress. Additionally, studies should investigate whether modifying the psychoacoustic or acoustic properties of tinnitus or trigger sounds influences these beliefs and, in turn, alters the distress pathway. In other words, refining these models requires a better understanding of how the physical properties of tinnitus or trigger sounds interact with an individual’s cognitive and emotional responses. Future research is also needed to develop psychoacoustic measurements that can assess and identify the most troublesome physical or psychophysical properties of tinnitus or trigger sounds and explore whether their omission or modification of such components changes the distress pathway [172,173]. Future studies should investigate whether combining disorder-specific CBT models developed separately for tinnitus, hyperacusis, and misophonia could yield a more comprehensive framework for understanding distress pathways and resilience mechanisms, while also offering explanatory power for a range of phenomenological features observed across both clinical and nonclinical populations [174]. Incorporating neurobiological and physiological measures, such as neuroimaging and biomarkers of stress, could provide further validation of these models [175,176,177]. Additionally, research should examine how individual differences—such as personality traits [178], coping strategies [179], and comorbid conditions, including medical, psychological, or other hearing disorders [180,181]—influence the ability of CBT models to explain distress. The clinical use of these models should remain flexible, focusing on assessing whether they adequately explain a patient’s distress. Clinicians should be prepared to collaboratively modify the model with the patient as needed, ensuring it accurately represents individual experiences.

### 5.2. Further Research on CBT Outcomes

Research evidence supports CBT as a beneficial intervention for tinnitus, hyperacusis, and misophonia, with both psychologists and audiologists delivering effective treatments. Systematic reviews and RCTs indicate that psychologist-delivered CBT significantly reduces tinnitus distress, with ESs ranging from 0.44 to 0.7. Audiologist-delivered CBT has also shown promising outcomes, particularly in reducing tinnitus impact [110,112,113]. However, the reported ES of 2.6 for video-based audiologist-delivered CBT was derived from a pre–post design [113] rather than an RCT, highlighting the need for further controlled trials. In controlled studies, ESs represent the between-group differences in pre-to-post change scores, helping to isolate the specific impact of the intervention from placebo effects or natural recovery. In contrast, ESs from uncontrolled studies reflect within-group pre–post changes and are more susceptible to inflation due to non-specific factors such as spontaneous improvement or regression to the mean. As a result, ESs in uncontrolled studies are often larger than those observed in RCTs but are considered less robust and less reliable indicators of true treatment efficacy. ES values above 0.2 are considered small, those above 0.5 are considered medium, and those above 0.8 are considered large [182].

Despite this, audiologist-delivered CBT remains highly acceptable to patients, with nearly all participants rating it favourably [110]. iCBT guided by audiologists has demonstrated positive effects [119,122,125,126,127], while unguided iCBT has also shown benefits, though it is associated with higher dropout rates. Rodrigo et al. [183] applied exploratory data mining techniques, specifically decision tree models, to identify variables linked to treatment success in iCBT for tinnitus. Their analysis revealed that higher educational level, greater baseline tinnitus severity, and higher levels of depression were associated with an increased likelihood of achieving a successful outcome. These findings align with evidence from face-to-face CBT, where patients with higher anxiety levels and more severe tinnitus impact have been shown to benefit more from CBT compared to those with lower anxiety and milder tinnitus severity [184].

Direct comparisons between different provider types are currently lacking. Future research should evaluate whether CBT outcomes vary based on the provider, considering factors such as training, patient preference, and long-term effects. Additionally, incorporating neuroimaging (e.g., fMRI, EEG) and physiological measures in future RCTs could offer objective insights into the neural mechanisms underlying symptom improvement, ultimately helping to refine CBT interventions for tinnitus, hyperacusis, and misophonia.

### 5.3. Process Analysis and Support Strategies for CBT Non-Responders

CBT is considered a complex intervention due to its multiple interacting components, including education, cognitive restructuring, behavioural modifications, empathic listening, and the clinician effect [185]. Aazh et al. [110] examined patients’ views on the effectiveness of CBT components, finding that therapist empathic listening was rated the most effective, significantly outperforming other techniques. This aligns with another study where counselling received the highest mean score, followed by education, CBT, and hearing tests. Notably, only 6% of respondents rated counselling as 3/5 or below, compared to 9% for education, 12% for hearing tests, and 15% for CBT [186]. According to UK Medical Research Council guidance, evaluating such interventions requires process analysis to identify mechanisms of change, key active ingredients, contextual influences, and factors affecting implementation [185,187,188]. Qualitative research can also enhance the evaluation of complex interventions [189]. There are some good examples of using qualitative research to modify CBT treatment for tinnitus [107,190,191,192]. Future studies on CBT for tinnitus, hyperacusis, and misophonia should incorporate process analysis or qualitative methods to better understand the role of each therapy component and how they can be refined for improved outcomes.

Based on the studies reviewed, it is evident that while most patients who undergo CBT for tinnitus, hyperacusis, or misophonia experience a reduction in distress, not all achieve this outcome. Marks et al. [193] and Fuller et al. [109] both reported that despite undergoing CBT, a proportion of patients did not experience significant improvement in tinnitus distress or impact. Aazh et al. [113] found that even after CBT, some patients continued to experience severe tinnitus impact and difficulties related to hyperacusis and misophonia, indicating that not all benefited from the intervention. Similarly, Beukes et al. [122,125] reported that a substantial number of patients who received iCBT still had scores indicating the need for further treatment. In another study on unguided iCBT, Aazh et al. [129] found that some participants continued to experience anxiety and depression symptoms even after treatment. A precise quantitative summary of the proportion of individuals who continue to experience distressing symptoms after CBT was not possible due to variability in outcome reporting and lack of access to raw data; future meta-analytic research or large-scale studies using individual participant data would be valuable for establishing more accurate estimates. To support this, future trials should consistently report the number and proportion of participants who continue to experience clinically significant distress following treatment. Overall, research indicates that while CBT, including iCBT, is generally effective in reducing distress, a proportion of patients continue to experience persistent symptoms and may require ongoing support or additional therapeutic interventions. This raises an important question about the next steps for those who do not experience significant improvement. Currently, there is limited research on effective therapeutic interventions specifically for patients who do not respond adequately to CBT. Future studies should explore tailored strategies to support this subgroup, such as the potential benefits of extended or modified CBT, more intense psychiatric or psychological interventions, the integration of pharmacological treatments, or alternative therapeutic approaches such as various forms of sound therapy [40,194] or bimodal stimulation [195,196,197]. It is also important to consider that if CBT does not help this subgroup, it may be more appropriate to shift the focus from attempting further CBT to providing holistic care that accommodates their ongoing challenges [198,199]. Accepting that some individuals may continue to experience distress despite therapy allows for a more compassionate and realistic approach [200]. This may involve developing supportive care models that prioritise quality of life and adaptive strategies rather than continuously seeking to eliminate the distress caused by their symptoms [201].

### 5.4. Proportion of Individuals Needing CBT for Tinnitus, Hyperacusis and Misophonia

Between 64% and 83% of people with normal hearing thresholds report perceiving tinnitus-like sounds when sitting in a silent anechoic chamber, suggesting that some form of tinnitus can be considered a common experience rather than a disorder [202,203,204,205,206]. However, in these individuals, the tinnitus is short-lasting and typically disappears once they leave the silent environment and return to everyday settings. Typically, tinnitus is considered present when it lasts more than 5 min, and in the UK, approximately 10.1% of the population (1 in 10 people) experience tinnitus of this duration [151]. There are other studies on the prevalence of tinnitus in the UK but they are limited to a specific age range (i.e., 40–69 years old) [207,208].

Not everyone who experiences tinnitus is significantly affected by it. De Ridder et al. [209] defined tinnitus as the conscious awareness of a tonal or composite noise without an identifiable external acoustic source. It becomes a tinnitus disorder when it is linked to emotional distress, difficulties with thinking or concentration, or heightened physical stress responses, which can lead to changes in behaviour and problems with daily functioning. In a study by Davis and Refaie [151], it was reported that 0.5% of individuals stated that tinnitus severely impacted their lives. Often, epidemiological studies assess the impact of tinnitus by asking participants one or two multiple-choice questions. However, this method has significant limitations, and its results should be interpreted cautiously. To accurately assess the severity of tinnitus-related distress or its impact on an individual’s life, the use of validated psychometric instruments and in-depth clinical interviews is recommended [38,111,210,211]. A study by Aazh and Moore [111] also highlighted the limitations of validated psychometric instruments in diagnosing distressing tinnitus or severe impact that may require CBT. Fackrell et al. [212] also found that, in addition to using questionnaires for hyperacusis, thorough patient interviews are necessary to fully explore all potential issues and make informed treatment decisions. One known limitation of questionnaires is response bias, where factors such as the respondent’s expectations, interpretation of the questions, research participation effect, and emotional and motivational influences may affect the results [213,214,215]. Therefore, results from questionnaires about the severity of tinnitus/hyperacusis/misophonia distress should be interpreted with caution, considering their accuracy, including sensitivity, specificity, predictive values, differential item functioning, and likelihood ratios, especially in studies aimed at informing policymakers or guiding resource allocation in health and social care [216,217]. Aazh and Moore [111] proposed that an in-depth interview should be considered the “gold standard” for diagnosing distressing tinnitus or hyperacusis/misophonia. This approach can help distinguish between tinnitus-related distress and mere annoyance caused by symptoms that do not necessarily impact daily functioning or quality of life. Additionally, it can help differentiate distress directly linked to tinnitus or hyperacusis/misophonia from distress related to other psychological, medical, or socioeconomic factors. CBT for tinnitus is only needed when the distress is directly linked to tinnitus, not to other problems (the same applies to CBT for hyperacusis/misophonia). When the distress is caused by other issues, such as hearing loss, balance problems, depression, psychosis, or other underlying conditions, interventions targeting these underlying problems should be prioritised. This approach aligns with public health studies on illness severity distribution, which emphasise that ignoring comorbidities can lead to an overestimation of illness severity. This is particularly relevant for conditions that are more common in the elderly or for mental disorders where comorbidity with another mental or physical disorder is frequent [218]. In the context of tinnitus or tinnitus-related distress, it is crucial to account for the impact of common comorbidities, such as hearing loss [181] or mental illness [219], to avoid overestimating the severity of tinnitus itself.

Our analysis shows that around 133,000 people in the UK (1 in 504) may need CBT for tinnitus, indicating that severe cases are rare despite tinnitus being common. This highlights the need to update information shared with patients, charities, and healthcare providers. If severe impact is defined as the need for CBT, our analysis indicates that only 1 in 52 people with tinnitus experience severe impact. This is based on the assumption that when CBT for tinnitus is not needed, it is either because the tinnitus does not significantly impact the person’s life or because the distress they are experiencing is not actually caused by tinnitus but by other factors. In fact, most people with tinnitus are able to manage well, and in many cases, their difficulties may be linked to hearing loss or other underlying issues. Overestimating the prevalence of severe tinnitus requiring treatment can lead to several negative consequences. First, patients diagnosed with tinnitus may become unnecessarily anxious, fearing that their condition is more severe than it actually is. For example, believing that one in six people with tinnitus will experience significant distress may increase stress and exacerbate symptoms, creating a self-fulfilling prophecy. Additionally, healthcare providers may encounter an influx of patients seeking treatment for tinnitus that does not warrant intervention, placing a strain on resources and increasing healthcare costs. Moreover, if the prevalence of severe tinnitus is overestimated, research funding may become disproportionately focused on severe cases, diverting attention from prevention, early intervention, or milder forms of tinnitus. This imbalance can limit the availability of resources for those who might benefit from early support. Patients may also undergo unnecessary treatments—such as medications or invasive procedures—that may cause side effects and increase healthcare costs. Exaggerating the severity of tinnitus can create fear, making the condition seem more debilitating than it typically is. This perception may discourage individuals with mild symptoms from managing their condition through lifestyle changes and coping strategies. Additionally, misrepresenting the prevalence can influence insurance policies and healthcare planning, leading to increased costs or policy changes that do not accurately reflect the true burden of the condition.

Similar principles apply to misophonia and hyperacusis, where severity can vary widely. At the extreme end, these conditions can be debilitating, while at the other end, they may represent less common presentations of natural human variation that do not require intervention [203,220]. Sensitivity to certain sounds is relatively common, but it does not necessarily indicate a disorder such as hyperacusis or misophonia. While approximately half of the population reports being moderately noise-sensitive, only a small fraction meets the clinical criteria for hyperacusis or misophonia. Research indicates that although many people find certain sounds irritating, only those whose symptoms significantly impact their daily lives might require CBT.

In summary, tinnitus, hyperacusis, and misophonia are classified as tinnitus disorder or clinical hyperacusis/misophonia when they cause significant distress and impairment in social, occupational, recreational, or other daily activities [59,186,209,221]. This paper focuses on these clinical conditions and emphasises the need for further research to develop methods that can effectively distinguish between clinical and subclinical presentations.

## 6. Conclusions

CBT has consistently proven to be an effective treatment for managing tinnitus, hyperacusis, and misophonia, with both psychologist- and audiologist-delivered approaches demonstrating significant improvements in reducing distress. iCBT also shows promise, especially when guided by professionals, offering a more accessible option for patients. Despite its demonstrated efficacy, it is important to note that not all patients who undergo CBT experience sufficient relief, raising critical questions about the best course of action for those who do not respond to standard CBT protocols.

Our analysis suggests that approximately 133,000 people in the UK (1 in 504) may require CBT for tinnitus, indicating that severe cases are relatively uncommon despite tinnitus being a widespread condition. This highlights the importance of accurately assessing the prevalence of severe tinnitus to ensure appropriate resource allocation and patient support.

Future research should aim to identify factors that influence CBT treatment outcomes, particularly for individuals who do not respond to conventional approaches. Direct comparisons between psychologist- and audiologist-led interventions are necessary to optimise collaboration and develop tailored treatment strategies. Additionally, hybrid therapy models that combine digital and face-to-face approaches warrant exploration, as they could enhance both accessibility and effectiveness.

Long-term follow-up studies are essential to assess the sustainability of CBT outcomes, while further research into iCBT, particularly for hyperacusis and misophonia, could expand therapeutic options for patients. More randomised controlled trials (RCTs) are needed to evaluate various CBT methods and their efficacy for hyperacusis and misophonia specifically. Additionally, investigating neurobiological mechanisms and exploring the potential of combining CBT with pharmacological treatments could provide valuable insights into improving therapeutic success.

Ultimately, personalised care models and holistic approaches that accommodate those with persistent symptoms are vital for enhancing patient well-being. Addressing the diverse needs of individuals with tinnitus, hyperacusis, and misophonia will require ongoing research, collaboration among healthcare professionals, and a commitment to refining and expanding treatment approaches.

## Figures and Tables

**Table 1:** Examples of different components in the transdiagnostic CBT model. The table is based on findings from various research studies; however, the examples are synthesised using three hypothetical patients—one with tinnitus, one with hyperacusis, and one with misophonia.

	Tinnitus	Hyperacusis	Misophonia
Patient	Jack (45-year-old man)	Sarah (30-year-old woman)	Saman (25-year-old man)
Trigger	Tinnitus perception	Certain everyday sounds (e.g., traffic noise [83])	Sounds associated with oral functions (e.g., chewing) [17]
Initial emotion	Irritated [79]	Anxiety [83]	Anger [59]
Initial bodily sensations	Aural fullness as a result of muscle spasms in middle ear [84]	Aural pain [83] as a nociceptive reflex [85,86], spasm of tensor tympani and stapedial tendons [87], flutter/middle ear myoclonus [88]	Autonomous sensory meridian response (ASMR) [89]
Initial behaviour	Rubbing ears or changing position [79]	Startle response [83]	Spontaneous mimicry [90]
Automatic thoughts and their appraisals	“I can no longer cope with tinnitus” [34], “my tinnitus will lead to a nervous breakdown” [56] “I should avoid unpleasant situations at all cost” [79]	“My sound-related pain handicaps me”, “The longer I am exposed to sound, the worse my hyperacusis symptoms become”, “I must control the environment around me to reduce my hyperacusis symptoms” [83,91]	“I will lose control” [92], “This is unbearable” [93], “They are selfish” [59]
Follow-on emotion	Fear [82]	Feeling depressed [83]	Feeling guilty [92]
Follow-on bodily sensations	Tension or palpitation [79] or tension headaches [94,95,96]	Tension in muscles [83] or headache [88]	Increased muscle tension and tightness or discomfort in the stomach [93]
Follow-on behaviour	Avoiding silence to prevent tinnitus from becoming noticeable [74]	Avoid exposure to sounds, use of hearing protection [83]	Avoidance or locating the sound source [20]
More evaluative thoughts	“I will be isolated” [62]	“I am not normal” [62] “No one understands me” [83]	“I am inadequate” [93] “I am a bad person underneath” [59]

## Data Availability

The original contributions presented in this study are included in the article. Further inquiries can be directed to the corresponding author.
